# Flexible Wearable Pressure Sensor Based on Collagen Fiber Material

**DOI:** 10.3390/mi13050694

**Published:** 2022-04-28

**Authors:** Zhiqing Peng, Shijie Zheng, Xia Zhang, Junlong Yang, Shizhou Wu, Chen Ding, Lei Lei, Lei Chen, Guoying Feng

**Affiliations:** 1 Institute of Laser & Micro/Nano Engineering, College of Electronics and Information Engineering, Sichuan University, Chengdu 610065, China; zh1q1ng_peng@stu.scu.edu.cn (Z.P.); zhengshijie@stu.scu.edu.cn (S.Z.); zhangxia1@stu.scu.edu.cn (X.Z.); 2 State Key Laboratory of Polymer Materials Engineering of China, Sichuan University, College of Polymer Science and Engineering, Chengdu 610065, China; yangjl@scu.edu.cn; 3 West China Hospital, Sichuan University, Chengdu 610041, China; dingchen0311@wchscu.cn (C.D.); leilei@scu.edu.cn (L.L.); chenlei@wchscu.cn (L.C.)

**Keywords:** activities signal, collagen fiber material, flexible wearable pressure sensor, capacitance detection circuit

## Abstract

Flexible wearable pressure sensors play a pivotal role in healthcare monitoring, disease prevention, and humanmachine interactions. However, their narrow sensing ranges, low detection sensitivities, slow responses, and complex preparation processes restrict their application in smart wearable devices. Herein, a capacitive pressure sensor with high sensitivity and flexibility that uses an ionic collagen fiber material as the dielectric layer is proposed. The sensor exhibits a high sensitivity (5.24 kPa^−1^), fast response time (40 ms), long-term stability, and excellent repeatability over 3000 cycles. Because the sensor is resizable, flexible, and has a simple preparation process, it can be flexibly attached to clothes and the human body for wearable monitoring. Furthermore, the practicality of the sensor is proven by attaching it to different measurement positions on the human body to monitor the activity signal.

## 1. Introduction

**Figure 1:**
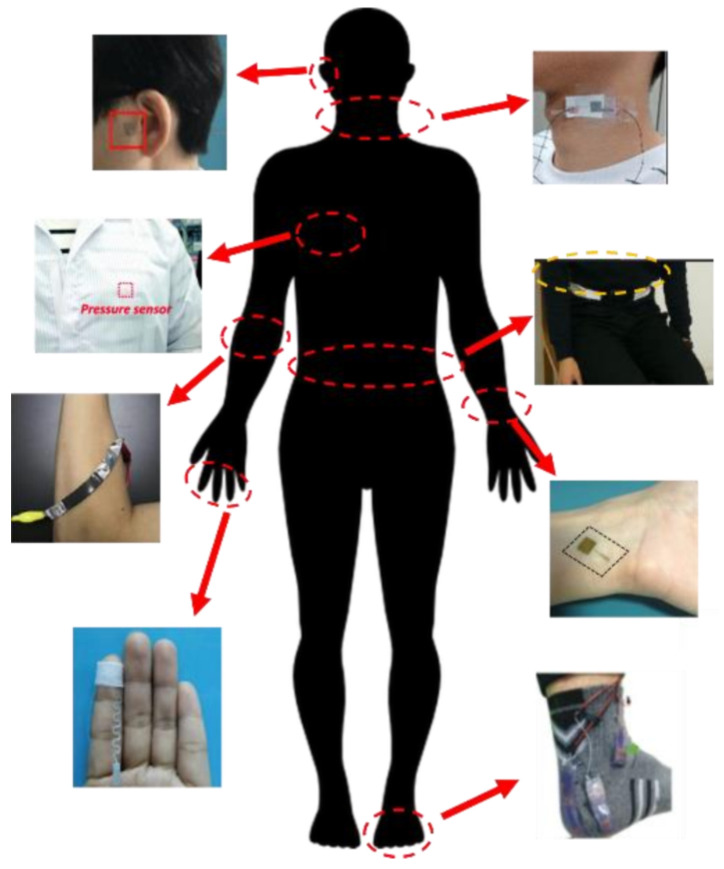
The detection positions for wearable pressure monitoring, such as ears [6], neck [10], arms [13], waist [14], wrists [15,19], fingers [16], chest [17], and feet [18].

Flexible wearable sensors are devices that can be worn and attached to different positions on the human skin to monitor various biological signals continuously without affecting the normal activities of the wearer. Flexible wearable pressure sensors exhibit a huge development potential in humanmachine interaction, virtual reality, healthcare monitoring, and disease diagnosis owing to their miniaturization and skin-friendliness. These sensors can continuously monitor pulse signals to prevent cardiovascular diseases [1,2,3,4,5,6,7], be used for developing respiration monitoring systems to prevent respiratory disorders such as sleep disorders and asthma [8,9,10], and closely detect tiny human activities to ensure early detection of abnormal motion control diseases such as Parkinson’s disease [11,12,13]. The detection positions of the reported wearable pressure sensors mainly include the ears [6], neck [10], arms [13], waist [14], wrists [15], fingers [16], chest [17], and feet [18], as shown in Figure 1.

Due to the complexity of human signals, it is important to effectively distinguish multiple mechanical stimuli for kinesthetic recognition [20]. Moreover, these sensors are dominated by flexible electronics that can transduce mechanical motion signals into electrical signals based on the piezoresistive, piezoelectric, triboelectric, and capacitive sensing mechanisms [14,15,21,22]. In contrast, capacitive pressure sensors have garnered significant attention owing to their simple and compact structure, low cost, and low power consumption [23]. Typically, capacitive pressure sensors are composed of two parallel electrodes with a conductive dielectric layer sandwiched between them. Therefore, the material of the dielectric layer determines the sensor performance. Wearable capacitive sensors still face several challenges in terms of energy supply, signal detection, and wearability. The recently developed triboelectric nanogenerator has provided ideas for the fabrication of self-powered wearable sensors [24,25]; moreover, selecting an appropriate detection position significantly improves the sensing signal strength. In addition, the wearability of sensors is also important for continuous wearable monitoring, which requires these sensors to be flexible and have low biotoxicity, and the dielectric layer material is the key for flexible stretchable sensors [26,27].

Collagen is an important and abundant protein in the animal kingdom, accounting for 25–30% of the mammalian body protein content [28]. Collagen fiber materials (CFMs), which exhibit outstanding biodegradability, good flexibility, and low toxicity, are recognized as excellent biological materials. They exhibit great compatibility with the human skin, as supported by several reported successful cases. In clinical medicine, pigskin collagen is used as a temporary biological skin substitute for burned skin. Previously, dog-skin collagen was used as a carrier for black plasters, owing to its high strength and warmth retention. In tissue engineering and other modern fields, collagen-based scaffolds aid in bone regeneration, cartilage repair, and bone reconstruction. In brief, the advantages of excellent skin-conformability, skin-friendliness, and natural stretchability make CFMs promising candidates for wearable sensors.

Herein, we propose a capacitive pressure sensor, with the advantages of a simple structure, good flexibility, and low cost, using an ionically charged CFM as the dielectric layer. Owing to the simple and efficient fabrication process of the dielectric layer material, the CFM-based capacitive pressure sensor exhibits the potential for large-scale production. We analyzed the electrical and mechanical properties of the CFM before and after immersion in an ionic liquid, studied the sensing performance of the sensor, and verified the detection feasibility of the sensor in wearable devices. The sensor has potential applications in wearable and continuous activity signal monitoring. It can be believed that our flexible wearable pressure sensor based on collagen fiber material can be integrated into various medical items for clinical applications. In pressure injury prevention, our novel material has a potential to sense, monitor, and alert abnormal pressure distribution as a film in cotton to decrease the possibility of maskne in the COVID-19 pandemic or as a layer in a cushion, which could significantly reduce the incidence of pressure damage and associated nursing workload for immobile and bedridden patients [29,30]. For diagnosis, our flexible force sensor-based material could be developed as a measuring device for the occlusal force distribution in malocclusion evaluation [31]. During orthodontic treatment, our flexible pressure sensor material could be modified as a part of and implanted into the braces to detect the force changes with clinical outcomes [32]. In treatment, pressure sensors can offer more accurate mechanical statistics than traditional experience and skill. For many podiatric disorders such as diabetes and a plantar forefoot ulcer, our sensor-based material could be designed as a collagen fiber pad for biomechanics understanding [33]. Further interventions including insoles and other foot orthoses could also be designed to correct and reduce pathological pressure in those patients.

## 2. Materials and Methods

### 2.1. Materials and Reagents

The ionic liquid, 1-butyl-3-methylimidalium-hexafluorophosphate ([BMIM]·PF_6_) was purchased from Adamas-Beta Co., Ltd. (Shanghai, China). Sodium formate, formic acid, ethylene glycol, sodium bicarbonate, magnesium oxide, and deionized water were purchased from ChengDu Chron Chemicals Co., Ltd. (Sichuan, China). A commercial zirconium complex tanning agent (commercial grade) was obtained from Sichuan Tingjiang New Material Co., Ltd. (Sichuan, China).

### 2.2. Preparation of CFM

**Figure 2:**
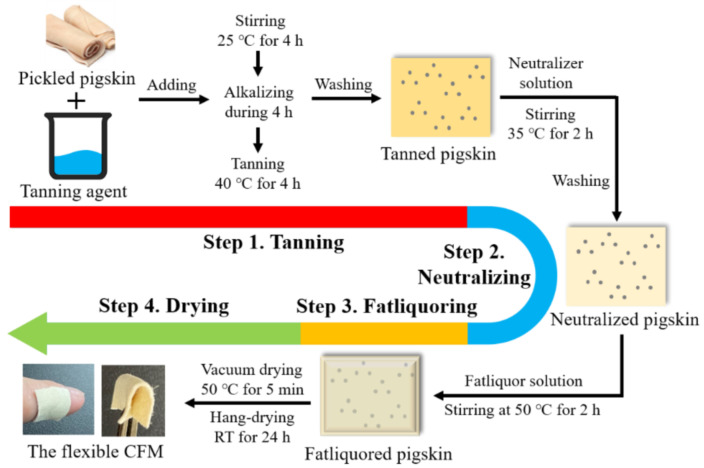
Preparation process of the pigskin CFM.

The preparation of CFM refers to industrial tanning technology, and the consumption of reagents used in the preparation process was calculated based on the weight of the pigskin. Pickled pigskin, used as the raw material for the preparation of the CFM, was supplied by a local tannery in China. As shown in Figure 2, the entire preparation process includes four steps: tanning, neutralization, fatty liquoring, and drying.

First, the pigskin was pretreated with 8.0% commercial zirconium complex tanning agent solution at 25 °C for 4 h; then, the 1.2% MgO was slowly added to the tanning agent solution to increase alkalinity (pH of the solution was basified to 3.8 for 4 h). Thereafter, the pigskin was tanned at 40 °C for 2 h and subsequently washed for further use. Second, the tanned pigskin was treated with a mixed solution of 2.0% HCOONa and 1.5% NaHCO_3_ until its pH was neutralized to 6.3, and the pigskin was washed for 30 min for further use. Third, the neutralized pigskin was treated with fatliquor solution at 50 °C for 2 h, after which the pH of fatliquor solution was adjusted to 4.0 with 1.0% formic acid; thereafter, the pigskin was washed before further use. Finally, the fatliquored pigskin was dried in vacuum at 50 °C for 5 min and subsequently hang-dried at room temperature (10–25 °C) for 24 h. Flexible CFM was obtained after staking.

### 2.3. Fabrication and Mechanism of Pressure Sensors

**Figure 3:**
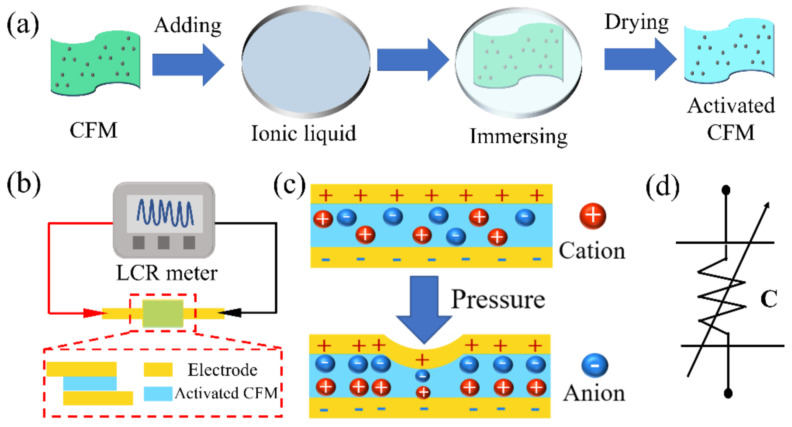
(**a**) Fabrication of the dielectric layer of the ionic liquid-based CFM, (**b**) schematic of the pressure sensor structure and its sensing system, (**c**) schematic illustration of the sensing principle, and (**d**) equivalent sensing of the pressure sensor.

The pressure sensor consisted of two Cu electrodes and a layer of fabric containing an ionic liquid with a capacitive-type structure. As shown in Figure 3a, the fabric, as the dielectric layer of the sensor, was immersed in the ionic liquid for 10 min at room temperature and subsequently hang-dried at room temperature until further use. Because CFM is a high load that can confine the ionic liquid into the microstructures of the fiber, and the ionic liquid is nonvolatile and exhibits a low toxicity, the material is low toxic and flexible; thus, the sensor is suitable for wearable sensing applications. In addition, the preparation process of CFM is simple and mature; it can achieve low-cost and large-scale industrial production, and the ion modification method and sensor structure are simple, so the sensor is expected to achieve mass production.

The structure and schematic of the pressure sensor and its sensing system are shown in Figure 3b. The sensor is triple-layered; the thickness and area of the dielectric layer are approximately 0.5 mm and 10 mm × 5 mm, respectively, and the relative area of the electrode is 10 mm × 5 mm. The pressure applied to the sensor could cause a change in capacitance, and the signals are recorded by an LCR meter such that the pressure sensing could be simply realized using the capacitance signals. The sensing mechanism is clearly explained in Figure 3c, and when external pressure is applied in the sensor, the electric double layer (EDL) of the sensor causes the change in capacitance. The contact area between the dielectric layer and the electrodes is proportional to the applied pressure, which is related to the EDL capacitance; thus, mechanical stimulation can be converted into capacitance signals by the sensor. The sensor can be considered as a structure with two EDL capacitors connected in series; the equivalent electrical circuit is shown in Figure 3d. Notably, the value of resistance (*R*) is not affected by pressure, and the capacitance is not sensitive to the resistance. Thus, the effect of resistance on the total capacitance can be ignored [16].

### 2.4. Instruments and Characterization

The mechanical properties of the materials were tested using a Gotech AI-7000 S servo control system universal testing machine (Taiwan, China). The capacitance of the sensor was detected using an LCR meter purchased from Renhe Technology Co., Ltd. (Zhejiang, China). Morphological characterizations of CFM before and after immersion in the ionic liquid were obtained using a Thermo Scientific Apreo scanning electron microscope (Waltham, MA, USA).

## 3. Results and Discussion

### 3.1. SEM Characterization of CFM

**Figure 4:**
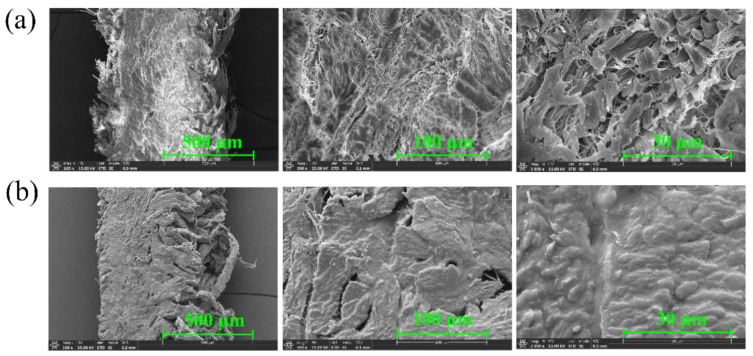
(**a**,**b**) SEM topography of the CFM (**a**) before and (**b**) after immersion in the ionic liquid.

Photomicrographs of the cross-section of CFM before and after immersion in the ionic liquid were observed using field-effect scanning electron microscopy (SEM), as shown in Figure 4a,b, respectively. The magnifications from left to right are 100×, 500×, and 2000×, respectively. CFM consists of collagen molecules with the triple helical structure; it is a three-dimensional network material with multilevel structure characteristics that is formed by the layer-by-layer assembly [34]. Notably, there are clear fibrous structures and interstices between the fibers before immersion in the ionic liquid, and these interspaces are filled after the immersion in the ionic liquid. This indicates that the ionic liquid is successfully loaded into the material.

### 3.2. Performance Characterization of the CFM

#### 3.2.1. Electrical Performance of the Dielectric Layer Materials

**Figure 5:**
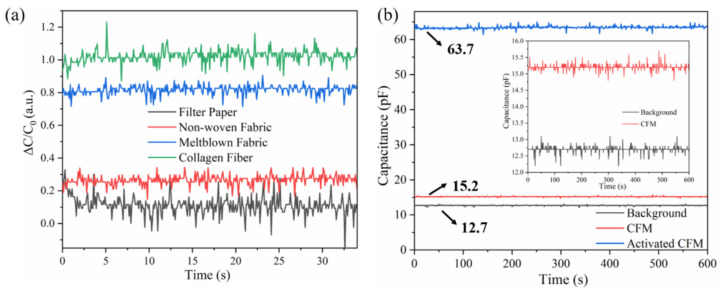
Comparison of (**a**) Δ*C*/*C*_0_ for the sensors with four different dielectric layers under the same pressure and (**b**) sensing response of the CFM before and after immersion in the ionic liquid.

The sensitivity *S* of the pressure sensor can be defined as follows:

(1) $$S=\delta \left(\Delta C{/C}_{0}\right)/\delta P$$

where *P* denotes the applied pressure on the sensor, and Δ*C* and *C*_0_ represent the capacitance before applying pressure and the change in capacitance after applying the pressure, respectively. It is observed that in the case of the same *P*, the sensitivity of the sensor is related to Δ*C* and *C*_0_, and it is determined by the ionic-activated material. According to the principle illustrated in Figure 3c, the flexibility and thickness of the material and its ion adsorption characteristic may affect the sensitivity of the sensor. In this study, we selected several common fabrics available in the market, such as the melt blown fabric, nonwoven fabric, and filter paper, for comparison with the CFMs. By measuring the capacitance before and after applying the same pressure to the sensors and calculating and normalizing the data, Figure 5a was obtained. The results clearly demonstrate that the filter paper and nonwoven fabric are difficult to use as the dielectric layer of the sensor owing to a lack of flexibility, and theoretically, the sensor using the CFM exhibits the highest sensitivity. 

To verify the effectiveness of the ionic liquids for the sensor, the common and activated CFMs were used in the control experiment, and the corresponding results are shown in Figure 5b. The capacitance (*C*) formula is as follows:

(2) $$C=\frac{\varepsilon \cdot S}{4\pi kd}$$

where *ε* is the dielectric constant, *S* is the area facing the two Cu electrodes, *k* is the electrostatic force constant, and *d* is the dielectric layer thickness. In theory, *C* is affected by *ε*, *S*, and *d*, and the application of an external pressure decreases *d* and results in an increase in *C*. Herein, *S* = 0.5 mm^2^, *d* = 0.6 mm, and *ε* of fabric before and after immersion in the ionic liquid is 3.39 and 63.7, respectively, indicating that the dielectric constant is significantly increased under these conditions. 

#### 3.2.2. Mechanical Properties of the Dielectric Layer Materials

**Figure 6:**
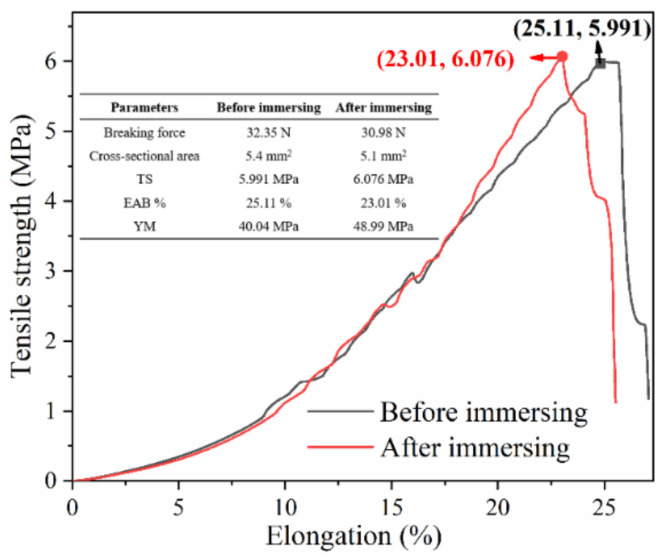
Comparison of the mechanical properties of the CFM before and after immersion in the ionic liquid.

To explore the influence of ionic liquids on the mechanical properties of CFM, the tensile strength (TS), elongation at break (EAB), and Young’s modulus (YM) of the CFM before and after the ionic loading were studied. The effective stretch area of the CFM was a rectangle with an area of 50 mm × 10 mm, and the stretching speed of the servo control system was 1.668 mm/s. All the parameters were calculated using the following equations [35]:

(3) $$\mathrm{TS}=\frac{F_{\max}}{\mathrm{Thickness}\times \mathrm{width}}$$

(4) $$\mathrm{EAB}\%=\frac{L-L_{0}}{L_{0}}\times 100$$

(5) $$\mathrm{YM}=\frac{F/A}{\Delta L/L_{0}}=\frac{F\cdot L_{0}}{A\cdot \Delta L}$$

where *F*_max_ denotes the breaking force of the material, *A* is the cross-sectional area (the product of thickness and width), *L* and *L*_0_ represent the length of the material when it breaks and its initial length, respectively, *F* is the tensile force on the material, and Δ*L* is the change in length. The relationship between TS and elongation for the material before and after immersion in the ionic liquid is shown in Figure 6. Evidently, the TS is 5.991 and 6.076 MPa, the *EAB*% are 25.11% and 23.01%, and the YM values are 40.04 and 48.99 MPa. These results demonstrate that no significant differences between the mechanical properties were observed, which explains why the mechanical properties of the CFM are almost unaffected by the ionic liquid load. 

### 3.3. Sensing Properties of the Sensor

#### 3.3.1. Sensitivity of the Sensor

**Figure 7:**
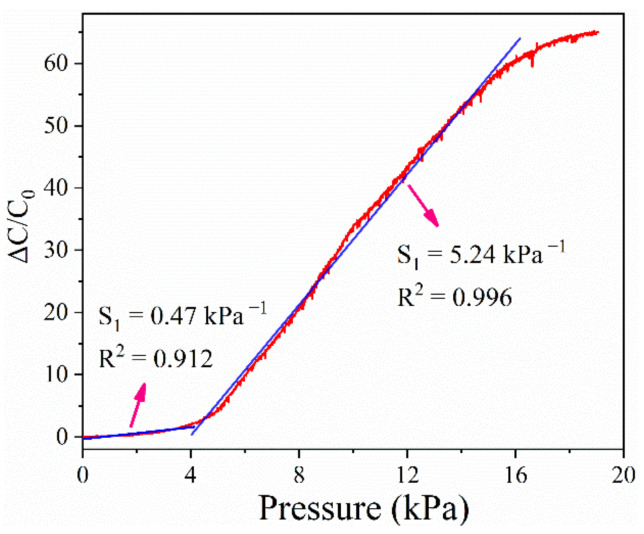
Response curves of pressure sensors under different pressures.

The capacitance of the sensor continues to increase with a continuous increase in the pressure applied to the sensor. Figure 7 shows the relationship between Δ*C*/*C*_0_ and pressure, wherein the slope of the curve yields the sensitivity of the sensor according to Equation (1). The sensor exhibits a linear sensitivity of 5.24 kPa^−1^ in the pressure range of up to 16 kPa. In the absence of external pressure, the distance between the dielectric layer and the electrode is microscale, and the contact area and capacitance are very small. The contact area between the dielectric layer and the electrode increases with the external pressure, and when the pressure increases to a critical value, the capacitance of the sensor is mainly affected by the deformation of the dielectric layer. Therefore, the sensor has different sensitivities in different pressure ranges.

#### 3.3.2. Response and Recovery of the Sensor

**Figure 8:**
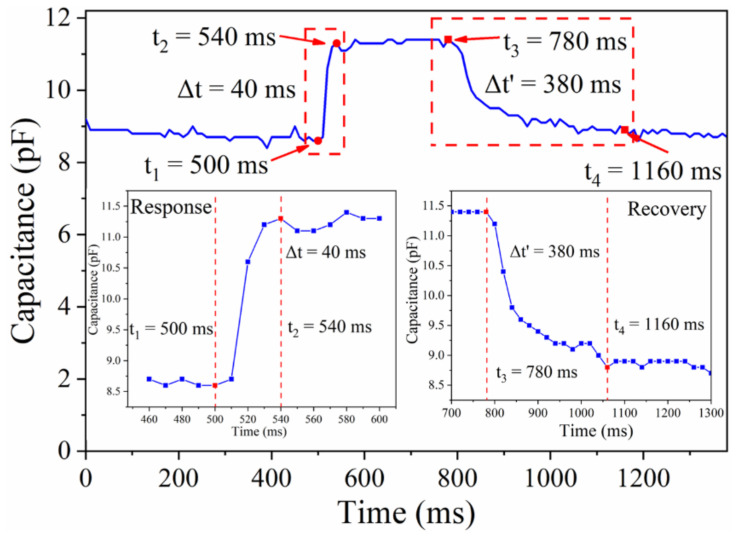
Response and recovery time of pressure sensor.

Figure 8 shows that the sensor exhibits a rapid increase in the capacitance within 40 ms, and the response time of the sensor is comparable to that of the biological skin (30–50 ms) [36]. Furthermore, the capacitance of the sensor recovered to its initial value within 380 ms after the load is removed. The longer recovery time is caused by the slow rebound of the CFM.

#### 3.3.3. Stability of the Sensor

**Figure 9:**
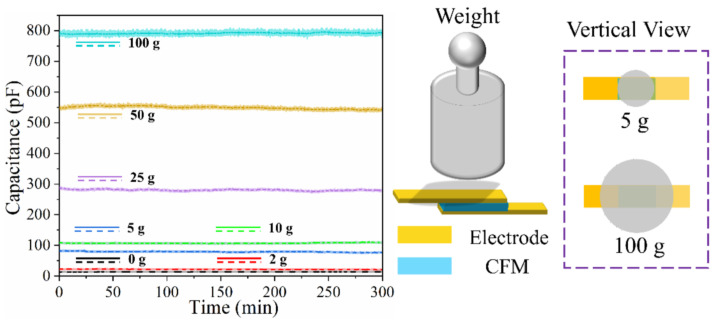
Long-term stability of pressure sensors to different pressures.

High stability is crucial for continuous-monitoring wearable sensors. The sensor was fixed on a horizontal table, and gravity was applied via weights in a natural state. The capacitance of the sensor was recorded within 5 h (sampling frequency of 10 Hz) under different pressures. The results, shown in Figure 9, demonstrate that the capacitance of the sensor remains stable for a long duration under different pressures. Because of the different contact areas between the weight and the sensor (as shown in the vertical view), the pressure applied on the sensor has a nonlinear relationship with the masses of the weights; thus, the value of capacitance has no reference significance.

#### 3.3.4. Repeatability of the Sensor

**Figure 10:**
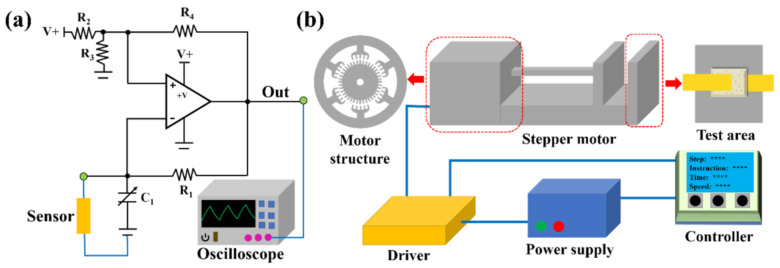
Schematic of the (**a**) capacitance detection circuit and (**b**) pressure application systems.

Owing to the low sampling frequency of the LCR meter, sensing data with high-frequency changes may not be accurately obtained. In this study, a real-time and rapid capacitance detection and matching pressure application systems were designed independently. Figure 10a,b show the control circuit diagram based on 555 timers and the drive concept diagram of the stepper motor, respectively.

The control circuit realizes a square-wave output via the charging and discharging of capacitor *C*_1_ by the resistor *R*_1_, and the oscillation period is determined by the time constant *R*_1_*C*_1_. When the sensor is connected to the circuit, the oscillation frequency (*f*) of the circuit can be expressed as follows:

(6) $$f=\frac{1}{T}=\frac{1}{K\cdot R_{1}\cdot \left(C_{1}+C\right)}$$

where *T* denotes the oscillation period, *K* is a constant determined by the resistor network, and *C* is the sensor capacitance. According to Equation (6), the oscillation frequency, *f*, is negatively correlated with the sensor capacitance, *C*. Therefore, *f* can also be used as the sensing signal of the sensor to detect the applied pressure. In addition, the pressure on the sensor is affected by the movement of the stepper motor. When the controller sends a movement signal to the stepper motor, the pressure applied to the sensor changes, and the sensing signal is recorded by the detection system. In this process, each movement signal (pressure signal) corresponds to a sensing signal, which is wirelessly transmitted to the computer in real time.

**Figure 11:**
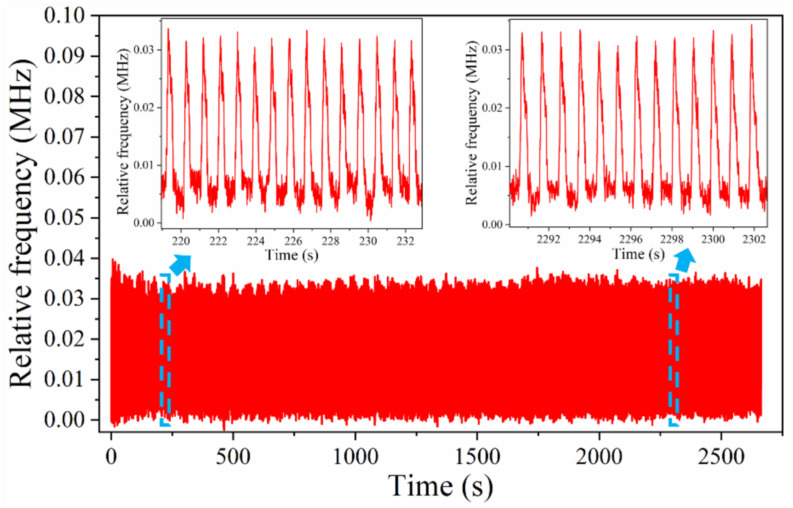
Repeatability of the flexible pressure sensor.

Figure 11 shows the frequency response curve of the sensor for approximately 3000 cycles of pressure application–relaxation. Although the temperature drift of the detection system caused a continuous increase in the frequency, the changes in the frequency for the pressure application–relaxation were equal, indicating that the sensor still exhibited a good response in the repeated experiments. The results demonstrate that the sensor can be effectively used to develop wearable devices and is expected to output physiological signals as well as achieve continuous human–motion monitoring. A video of the pressure application–relaxation is provided in the Supplementary Materials Video S1.

Table 1 summarizes the sensitivity, response time, sensing range, and cycle response time of some capacitive pressure sensors and compares them with the proposed pressure sensors. The pressure sensor proposed in this work not only exhibits good sensing performance, but also has low-cost and simple fabrication process.

### 3.4. Applications of the Sensor

**Figure 12:**
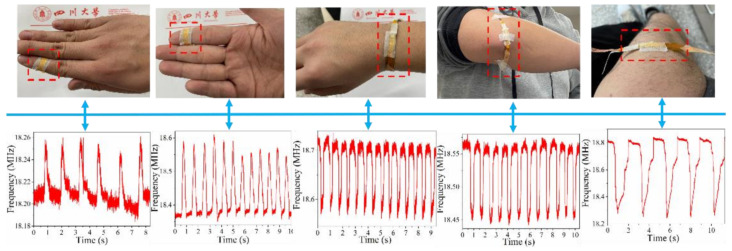
Measurement positions on the human body and the corresponding detection signals. From left to right: outside and inside of finger joints, wrist, elbow joint, and knee joint.

A simple experiment was conducted to record the signal and investigate the activity at different measurement positions on the human body. As shown in Figure 12, the sensor can be laminated on finger joints (inside and outside), wrists, elbow joints, and knee joints, and the activity signals of these parts, such as bending, can be captured as electric signals. The different waveform intervals represent the time difference of the activity; the different waveform intensities represent the strength of the activity, and the wave crest and trough represent the application and release of the pressure acting on the sensor. For the movement of the finger joints, the bending of the joint reduces the pressure on the sensor; the oscillation frequency increases, and the capacitance decreases. For the movement of the wrist, elbow, and knee, the bending of the joint increases the pressure on the sensor; the oscillation frequency decreases, and the capacitance increases. The ability to discriminate human activity verifies the feasibility of the sensor as a wearable device capable of medical diagnosis. Videos of the captured electrical signals are provided in Supplementary Materials Videos S2–S5, and the detectors are pasted to the elbow, wrist, and finger.

## 4. Conclusions

In conclusion, a wearable pressure sensor, which integrates the advantages of CFM and capacitive sensing, was developed for continuously monitoring pulse signals. The foremost advantages of the CFM are its flexibility, biocompatibility, and low biotoxicity, which determine its application potential in the field of wearable sensors. The sensitivity of the sensor was approximately 5.24 kPa^−1^. The wearable sensor exhibited long-term stability and showed excellent repeatability over 3000 cycles of pressure application–relaxation, with response and recovery times of approximately 30 and 380 ms, respectively, and successfully detected the human motion signal. Although the experiment and analysis are slightly crude, our results indicate that the sensor can be used to develop wearable devices owing to its excellent mechanical properties and sensing performance. The sensor is anticipated to be promising for healthcare monitoring and clinical diagnosis, owing to its simple preparation, low cost, great biocompatibility, and excellent flexibility. In the future, the introduction of the self-powered system and the integration of the sensing system will be considered, and the sensitivity of the sensor will be improved to expand monitoring applications.

## Figures and Tables

**Table 1:** Comparison of the proposed capacitive pressure sensor with reported sensor.

Sensitivity	Response Time	Sensing Range	Cycle Response Time	Ref.
17.5 kPa^−1^	90 ms	0–120 kPa	>6000	[9]
1.12 kPa^−1^	10 ms	0–40 kPa	>10,000	[10]
13.5 kPa^−1^	30 ms	0–175 kPa	>5000	[16]
0.16 kPa^−1^	70 ms	0–10 kPa	>10,000	[17]
2.51 kPa^−1^	84 ms	>10 kPa	>5000	[37]
3.13 kPa^−1^	94 ms	0–50 kPa	>5000	[38]
0.43 kPa^−1^	33 ms	0–50 kPa	>1000	[39]
5.24 kPa^−1^	40 ms	0–16 kPa	>3000	This work

## Data Availability

The data presented in this study are available on request from the corresponding author. The data are not publicly available due to restrictions e.g., privacy or ethical.

## Supplementary Materials

The following supporting information can be downloaded at: www.mdpi.com/article/10.3390/mi13050694/s1, Video S1: Pressure application–relaxation; Video S2: Dynamic monitoring of elbow bending; Video S3: Dynamic monitoring of wrist bending; Videos S4 and S5: Dynamic monitoring of finger bending.

## References

[1] Chu Y., Zhong J., Liu H., Ma Y., Liu N., Song Y., Liang J., Shao Z., Sun Y., Dong Y. (2018). Human pulse diagnosis for medical assessments using a wearable piezoelectret sensing system. Adv. Funct. Mater..

[2] Sun Y., Zhang Z., Zhou Y., Liu S., Xu H. (2021). Wearable Strain Sensor Based on Double-Layer Graphene Fabrics for Real-Time, Continuous Acquirement of Human Pulse Signal in Daily Activities. Adv. Mater. Technol..

[3] Pang C., Koo J.H., Nguyen A., Caves J.M., Kim M.G., Chortos A., Kim K., Wang P.J., Tok J.B.H., Bao Z. (2015). Highly skin-conformal microhairy sensor for pulse signal amplification. Adv. Mater..

[4] Wang J., Liu K., Sun Q., Ni X., Ai F., Wang S., Yan Z., Liu D. (2019). Diaphragm-based optical fiber sensor for pulse wave monitoring and cardiovascular diseases diagnosis. J. Biophotonics.

[5] Kaisti M., Panula T., Leppänen J., Punkkinen R., Tadi M.J., Vasankari T., Jaakkola S., Kiviniemi T., Airaksinen J., Kostiainen P. (2019). Clinical assessment of a non-invasive wearable MEMS pressure sensor array for monitoring of arterial pulse waveform, heart rate and detection of atrial fibrillation. NPJ Digit. Med..

[6] Meng K., Chen J., Li X., Wu Y., Fan W., Zhou Z., He Q., Wang X., Fan X., Zhang Y. (2019). Flexible weaving constructed self-powered pressure sensor enabling continuous diagnosis of cardiovascular disease and measurement of cuffless blood pressure. Adv. Funct. Mater..

[7] Park D.Y., Joe D.J., Kim D.H., Park H., Han J.H., Jeong C.K., Park H., Park J.G., Joung B., Lee K.J. (2017). Self-powered real-time arterial pulse monitoring using ultrathin epidermal piezoelectric sensors. Adv. Mater..

[8] Park S.W., Das P.S., Chhetry A., Park J.Y. (2017). A flexible capacitive pressure sensor for wearable respiration monitoring system. IEEE Sens. J..

[9] Kim Y.-R., Kim M.P., Park J., Lee Y., Ghosh S.K., Kim J., Kang D., Ko H. (2020). Binary Spiky/Spherical Nanoparticle Films with Hierarchical Micro/Nanostructures for High-Performance Flexible Pressure Sensors. ACS Appl. Mater. Interfaces.

[10] Jin T., Pan Y., Jeon G.-J., Yeom H.-I., Zhang S., Paik K.-W., Park S.-H.K. (2020). Ultrathin nanofibrous membranes containing insulating microbeads for highly sensitive flexible pressure sensors. ACS Appl. Mater. Interfaces.

[11] Chen X., Shao J., An N., Li X., Tian H., Xu C., Ding Y. (2015). Self-powered flexible pressure sensors with vertically well-aligned piezoelectric nanowire arrays for monitoring vital signs. J. Mater. Chem. C.

[12] Amit M., Chukoskie L., Skalsky A.J., Garudadri H., Ng T.N. (2020). Flexible pressure sensors for objective assessment of motor disorders. Adv. Funct. Mater..

[13] Yang G., Tian M.-Z., Huang P., Fu Y.-F., Li Y.-Q., Fu Y.-Q., Wang X.-Q., Li Y., Hu N., Fu S.-Y. (2021). Flexible pressure sensor with a tunable pressure-detecting range for various human motions. Carbon.

[14] Zhang H., Zhang J., Hu Z., Quan L., Shi L., Chen J., Xuan W., Zhang Z., Dong S., Luo J. (2019). Waist-wearable wireless respiration sensor based on triboelectric effect. Nano Energy.

[15] Dagdeviren C., Su Y., Joe P., Yona R., Liu Y., Kim Y.-S., Huang Y., Damadoran A.R., Xia J., Martin L.W. (2014). Conformable amplified lead zirconate titanate sensors with enhanced piezoelectric response for cutaneous pressure monitoring. Nat. Commun..

[16] Lin Q., Huang J., Yang J., Huang Y., Zhang Y., Wang Y., Zhang J., Wang Y., Yuan L., Cai M. (2020). Highly sensitive flexible iontronic pressure sensor for fingertip pulse monitoring. Adv. Healthc. Mater..

[17] Jeon G.-J., Yeom H.-I., Jin T., Kim J., Yang J., Park S.-H.K. (2020). A highly sensitive, stable, scalable pressure sensor based on a facile baking-inspired foaming process for a human–computer interface. J. Mater. Chem. C.

[18] Kim K., Choi J., Jeong Y., Cho I., Kim M., Kim S., Oh Y., Park I. (2019). Highly Sensitive and Wearable Liquid Metal-Based Pressure Sensor for Health Monitoring Applications: Integration of a 3D-Printed Microbump Array with the Microchannel. Adv. Healthc. Mater..

[19] Yang J., Liu Q., Deng Z., Gong M., Lei F., Zhang J., Zhang X., Wang Q., Liu Y., Wu Z. (2019). Ionic liquid–activated wearable electronics. Mater. Today Phys..

[20] Li X., Cao J., Li H., Yu P., Fan Y., Xiao Y., Yin Y., Zhao X., Wang Z.L., Zhu G. (2021). Differentiation of Multiple Mechanical Stimuli by a Flexible Sensor Using a Dual-Interdigital-Electrode Layout for Bodily Kinesthetic Identification. ACS Appl. Mater. Interfaces.

[21] Choong C.L., Shim M.B., Lee B.S., Jeon S., Ko D.S., Kang T.H., Bae J., Lee S.H., Byun K.E., Im J. (2014). Highly stretchable resistive pressure sensors using a conductive elastomeric composite on a micropyramid array. Adv. Mater..

[22] Zang Y., Zhang F., Di C.-a., Zhu D. (2015). Advances of flexible pressure sensors toward artificial intelligence and health care applications. Mater. Horiz..

[23] ACS Applied Materials & InterfacesAsghar W., Li F., Zhou Y., Wu Y., Yu Z., Li S., Tang D., Han X., Shang J., Liu Y. (2020). Piezocapacitive Flexible E-Skin Pressure Sensors Having Magnetically Grown Microstructures. Adv. Mater. Technol..

[24] Yi F., Zhang Z., Kang Z., Liao Q., Zhang Y. (2019). Recent advances in triboelectric nanogenerator-based health monitoring. Adv. Funct. Mater..

[25] Khandelwal G., Raj N.P.M.J., Kim S.-J. (2020). Triboelectric nanogenerator for healthcare and biomedical applications. Nano Today.

[26] Xu D., Cao J., Liu F., Zou S., Lei W., Wu Y., Liu Y., Shang J., Li R.-W. (2022). Liquid Metal Based Nano-Composites for Printable Stretchable Electronics. Sensors.

[27] Cao J.W., Liang F., Li H.Y., Li X., Fan Y.J., Yin Y., Li F., Xu J., Feng H., Xu D. (2021). Ultra-robust stretchable electrode for e-skin: In situ assembly using a nanofiber scaffold and liquid metal to mimic water-to-net interaction. InfoMat.

[28] Silvipriya K., Kumar K.K., Bhat A., Kumar B.D., John A., Lakshmanan P. (2015). Collagen: Animal sources and biomedical application. J. Appl. Pharm. Sci..

[29] Diomidous M., Dalamaga M., Nikolopoulos M., Tzortzis E., Stratigou T., Antonakos G., Karampela I. (2020). Wireless Monitoring Through Wearable Devices in the ICU: Are We Close?. Stud. Health Technol. Inform..

[30] Hadzavdic A., Bukvic Mokos Z. (2021). Maskne: A New Entity in the COVID-19 Pandemic. Acta Dermatovenerol. Croat. ADC.

[31] Lin K.R., Chang C.H., Liu T.H., Lin S.W., Lin C.H. (2011). Experimental and numerical estimations into the force distribution on an occlusal surface utilizing a flexible force sensor array. J. Biomech..

[32] Skaik A., Wei X.L., Abusamak I., Iddi I. (2019). Effects of time and clear aligner removal frequency on the force delivered by different polyethylene terephthalate glycol-modified materials determined with thin-film pressure sensors. Am. J. Orthod. Dentofacial Orthop..

[33] Panera-Rico E., Castillo-Lopez J.M., Palomo-Toucedo I.C., Chacon-Giraldez F., Ramos-Ortega J., Dominguez-Maldonado G. (2021). Use of Plantar Pressure Sensors to Take Weight-Bearing Foot Casts. Sensors.

[34] Xu H., Wang Y., Liao X., Shi B. (2020). A collagen-based electrolyte-locked separator enables capacitor to have high safety and ionic conductivity. J. Energy Chem..

[35] Wu J., Liu F., Yu Z., Ma Y., Goff H.D., Ma J., Zhong F. (2020). Facile preparation of collagen fiber–glycerol-carboxymethyl cellulose composite film by immersing method. Carbohydr. Polym..

[36] Chortos A., Bao Z. (2014). Skin-inspired electronic devices. Mater. Today.

[37] Zhang Z., Gui X., Hu Q., Yang L., Yang R., Huang B., Yang B.R., Tang Z. (2021). Highly sensitive capacitive pressure sensor based on a micropyramid array for health and motion monitoring. Adv. Electron. Mater..

[38] Ha K.H., Zhang W., Jang H., Kang S., Wang L., Tan P., Hwang H., Lu N. (2021). Highly Sensitive Capacitive Pressure Sensors over a Wide Pressure Range Enabled by the Hybrid Responses of a Highly Porous Nanocomposite. Adv. Mater..

[39] Luo Z., Chen J., Zhu Z., Li L., Su Y., Tang W., Omisore O.M., Wang L., Li H. (2021). High-resolution and high-sensitivity flexible capacitive pressure sensors enhanced by a transferable electrode array and a micropillar–PVDF film. ACS Appl. Mater. Interfaces.

